# Underrepresentation of racial and ethnic minorities in cascade testing for hereditary cancer syndromes

**DOI:** 10.1038/s41431-023-01364-2

**Published:** 2023-04-28

**Authors:** Muhammad Danyal Ahsan, Emily M. Webster, Natalie T. Nguyen, Murtaza Qazi, Sarah R. Levi, Lisa C. Diamond, Ravi N. Sharaf, Melissa K. Frey

**Affiliations:** 1 Weill Cornell Medicine, New York, NY USA; 2 Weill Cornell Medicine - Qatar, Doha, Qatar; 3 Memorial Sloan Kettering Cancer Center, New York, NY USA

**Keywords:** Genetics research, Public health

Cascade genetic testing is the process of extending genetic testing to at-risk blood relatives of individuals (called “probands”) found to carry cancer predisposition germline pathogenic gene variants. Cascade testing is critical as relatives found to carry a pathogenic gene variant have the opportunity to pursue targeted cancer risk reduction and early detection, decreasing cancer-associated morbidity and mortality [1, 2]. The Centers for Disease Control and Prevention Office of Public Health Genomics has designated cascade genetic testing as a tier one genomic application for Hereditary Breast and Ovarian Cancer and Lynch syndrome [3]. Mathematical modeling suggests that the combination of germline genetic testing at time of cancer diagnosis with subsequent cascade testing of at-risk relatives has the potential to identify all individuals with a cancer predisposing pathogenic variant in the United States in less than a decade [4]. However, our recent systematic review published in the Journal of Clinical Oncology demonstrates that only about a third of at-risk relatives undergo recommended cascade testing [5].

It is possible that there are differences in uptake of cascade testing between racial and ethnic groups; it is already known that there is disproportionate under-recognition of hereditary cancer syndromes amongst racial and ethnic minorities [6, 7]. To further assess, we performed a sub-analysis on studies included in our published systematic review [5]. Among 50 articles included for meta-analysis, only 12 (24%) publications reported demographic data for probands and only 9 (18%) for relatives. Among 1799 probands whose race and/or ethnicity was reported in these studies, 1388 (77.2%) self-identified as non-Hispanic White, 266 (14.8%) as Asian, 56 (3.1%) as Hispanic, 44 (2.4%) as Multiple, 23 (1.3%) as Black, 15 (0.8%) as Other, and 7 (0.4%) as American Indian/Alaska Native (Table 1). Among 2281 relatives whose race and/or ethnicity was reported, 1646 (72.2%) self-identified as non-Hispanic White, 318 (14.0%) as Asian, 193 (8.5%) as Hispanic, 58 (2.5%) as Black, 53 (2.3%) as Multiple, 11 (0.5%) as Other, and 2 (0.1%) as American Indian/Alaska Native (Table 1). Available data were insufficient to calculate summary estimates of cascade testing rates among racial and ethnic minorities nor quantitatively compare them to those of non-Hispanic Whites.

**Table 1:** Demographics of proband and relative populations among studies that reported demographic information.

Study	Proband Demographics	Relative Demographics
Bednar 2020	Non-Hispanic White: 140 (93.3%)	Not reported
	Black: 2 (1.3%)	
	Asian: 2 (1.3%)	
	Native American/Alaska Native: 2 (1.3%)	
	Other: 4 (2.7%)	
Biesecker 2000	Not reported	Non-Hispanic White: 172 (100%)
Caswell-Jin 2019	Non-Hispanic White: 697 (85.6%)	Non-Hispanic White: 899 (86.0%)
	Hispanic: 36 (4.4%)	Hispanic: 69 (6.6%)
	Black: 4 (0.5%)	Black: 5 (0.5%)
	Asian: 35 (4.3%)	Asian: 34 (3.3%)
	Native American: 1 (0.1%)	Multiple: 38 (3.6%)
	Multiple: 41 (5.0%)	
Courtney 2019	Asian: 175 (95.6%)	Asian: 106 (94.6%)
	Other: 8 (4.4%)	Other: 6 (5.4%)
Dilzell 2014	Non-Hispanic White: 41 (91.1%)	Non-Hispanic White: 20 (90.9%)
	Native American: 2 (4.4%)	Hispanic: 1 (4.5%)
	Black: 1 (2.2%)	Native American: 1 (4.5%)
	Asian: 1 (2.2%)	
Fehniger 2013	Non-Hispanic White: 32 (43.8%)	Non-Hispanic White: 135 (30.5%)
	Hispanic: 17 (23.3%)	Hispanic: 123 (27.8%)
	Asian: 14 (19.2%)	Asian: 117 (26.4%)
	Black: 7 (9.6%)	Black: 53 (12.0%)
	Multiple: 3 (4.1%)	Multiple: 15 (3.4%)
Finlay 2008	Non-Hispanic White: 114 (99.1%)	Not reported
	Other: 1 (0.9%)	
Griffin 2020	Non-Hispanic White: 62 (96.9%)	Not reported
	Black: 2 (3.1%)	
Hadley 2003	Non-Hispanic White: 87 (87.0%)	Not reported
	Black: 7 (7.0%)	
	Hispanic: 3 (3.0%)	
	Asian: 2 (2.0%)	
	Native American: 1 (1.0%)	
Lerman 1996	Not reported	Non-Hispanic White: 192 (100%)
Lerman 1999	Not reported	Non-Hispanic White: 138 (99.3%)
		Native American: 1 (0.7%)
Lieberman 2018	Non-Hispanic White: 148 (100%)	Not reported
McGivern 2004	Non-Hispanic White: 37 (97.4%)	Not reported
	Native American: 1 (2.6%)	
Petersen 2018	Non-Hispanic White: 30 (93.8%)	Non-Hispanic White: 90 (94.7%)
	Other: 2 (6.3%)	Other: 5 (5.3%)
Yoon 2011	Asian: 37 (100%)	Asian: 61 (100%)

In summary, the uptake of cascade genetic testing for hereditary cancer syndromes by racial and ethnic minority groups is not well elucidated. Among eight trials currently evaluating interventions for cascade testing registered on clinicaltrials.gov, six (75%) do not include the influence of race and ethnicity on uptake of cascade testing as a primary or secondary objective. The American Society of Human Genetics published guidance to improve representation of racial and ethnic minorities in genomics research, highlighting the need to recruit and engage historically disadvantaged and underrepresented communities [8]. Our findings indicate the need for studies of cascade testing within diverse populations.
